# Editorial: Advancements in drug development: exploring bi-specific antibodies as promising therapeutic strategies in oncology

**DOI:** 10.3389/fonc.2025.1651944

**Published:** 2025-07-08

**Authors:** Urs M. Weber, Julie M. Bailis, Angela Coxon, Sharon R. Pine, Giuseppe Giaccone

**Affiliations:** ^1^ Division of Medical Oncology, Department of Medicine, University of Colorado Anschutz Medical Campus, Aurora, CO, United States; ^2^ Oncology Research, Amgen Research, Amgen Inc., South San Francisco, CA, United States; ^3^ Oncology Research, Amgen Research, Amgen Inc., Thousand Oaks, CA, United States; ^4^ Department of Hematology and Medical Oncology, Weill Cornell Medical College, New York, NY, United States

**Keywords:** bi-specific antibodies, T-cell engager (TCE), NK cell engager (NKCE), drug development, oncology

Antibody-based therapies have revolutionized the treatment of cancer. Monoclonal antibodies (mAbs), particularly those targeting the PD-1/PD-L1 axis, are widely used across the field of oncology and have significantly improved treatment outcomes for many different malignancies. Bispecific antibodies (bsAbs) are part of the next wave of therapeutic innovation and provide added functionality. They can localize biological activity to a specific cell type or bring different cell types into close proximity of one another to enable a new activity. Immune engagers, such as T-cell engagers (TCEs) and natural killer cell engagers (NKCEs), are examples of the latter.

TCEs bind CD3 on T-cells and a tumor-associated antigen on tumor cells, thereby activating the T-cell and directing it to kill the tumor cells. The CD19xCD3 TCE, blinatumomab, showed efficacy in the treatment of B-cell malignancies and became the first TCE approved for the treatment of cancer in 2014. Since 2022, 6 additional TCEs have been approved for the treatment of hematologic malignancies, while only 2 have been approved for the treatment of solid tumors. NKCEs are designed to bind NK cells and tumor cells, activating NK cells to enhance tumor cell killing by antibody-dependent cellular cytotoxicity (ADCC). Several NKCEs are in preclinical and clinical development for the treatment of cancer, although none have yet been approved for commercial use. In this Frontiers Research Topic, *“Advancements in Drug Development: Exploring Bi-Specific Antibodies as Promising Therapeutic Strategies in Oncology,”* we explore the promise of TCEs and NKCEs, as well as the challenges that these treatments have to overcome.

Lloyd et al. discuss some of the challenges facing TCE development in solid tumors and suggest strategies for rational combinations to improve treatment efficacy. In preclinical syngeneic models, low numbers of baseline tumor-infiltrating T-cells, along with an immunosuppressive tumor microenvironment, can limit TCE efficacy as well as the durability of treatment response. Pre-treatment of mice with a vaccine or oncolytic virus increased T-cell infiltration into tumors, enabling effective TCE engagement and antitumor activity. For tumor models that contained T-cells at baseline, combinations that provided costimulatory T-cell signals, such as agonistic CD28 or 4-1BB antibodies, improved T-cell function and enhanced anti-tumor activity. The authors go on to introduce the idea of improving the duration of TCE treatment responses through the co-administration of vaccines to increase immunological memory or engage other immune cells to activate both innate and adaptive immunity. Deepening our understanding of the complex interactions between immune cells and tumor cells will be crucial if we are to continue to advance TCE therapy for solid tumors.

One of the main adverse events associated with TCE therapy is cytokine release syndrome (CRS). CRS is a byproduct of the TCE mechanism of action. While it can often be managed with premedication and stepwise dose escalation, CRS can cause serious complications for patients. Selvaggio et al. developed a computational model designed to predict the risk of CRS and identify potential additional mitigation strategies. They analyzed clinical and preclinical data and identified IL-6 and TNF-α as two cytokines that can be targeted to treat CRS without compromising TCE anti-tumor efficacy. The model also identified IFN-γ as a potential target relevant to CRS. The model provides a framework to study diverse factors that influence CRS development and to form hypotheses for CRS mitigation that can be studied clinically.

Another strategy to improve the efficacy and safety of TCEs is explored by (King et al.). They present original research that tests the hypothesis that TCE molecules that engage a specific subset of T-cells, Vγ9Vδ2 T-cells, can induce effective T-cell expansion and redirected T-cell-mediated cell lysis. They developed a novel TCE format that is comprised of a single domain antibody (VHH) that binds a tumor-associated antigen, and a bivalent VHH that has both a high affinity Vδ2-TCR and a low affinity Vδ2-TCR. Using TCEs directed towards different tumor antigens they demonstrated effective T-cell expansion and T-cell-dependent anti-tumor activity across preclinical models *in vitro*, *in vivo*, and *ex vivo*, charting a path towards improving the TCEs targeting Vγ9Vδ2 T-cells that previously made it to clinical testing but failed to advance further.

Finally, we include a review by Nikkhoi et al. on the current development and future potential of NKCEs. Much like with TCEs, many different NKCE configurations are being explored in an effort to optimize ADCC. The challenges are also similar, as tumor-associated NK cells may be insufficient in number or function for effective bsAb engagement. The authors propose strategies to improve NKCE treatment efficacy, including engineering them to enable additional cell killing mechanisms and targeting more than one tumor-associated antigen at a time to prevent treatment resistance. While the NKCEs are at an earlier stage in their development than the TCEs, they may yet mature into another treatment option for patients.

The use of immune engagers in various formats has vastly expanded the arsenal of available cancer treatments. The next wave of innovation represents a clear step forward in harnessing the capabilities of the immune system to kill tumor cells. The articles in this Frontiers Research Topic highlight some of the opportunities in and challenges to delivering TCEs and NKCEs to patients.

